# AGE DIFFERENCES IN PSYCHOSOCIAL AND HEALTH OUTCOMES AMONG ADULTS EXPERIENCING HOMELESSNESS

**DOI:** 10.1093/geroni/igz038.3543

**Published:** 2019-11-08

**Authors:** Carolyn L Turturro, Rosalie Otters, Zeferin Turturro

**Affiliations:** 1 University of Arkansas at Little Rock, School of Social Work, Little Rock, Arkansas, United States; 2 Auburn University, Auburn, Alabama, United States

## Abstract

Homelessness is a critical social problem for this nation. It takes a tremendous toll on individuals and families who live without stable shelter, adequate food, and personal security, as well as strains the societal resources. The mean age of death for people experiencing homelessness has been reported to be age 50, far less than the 76 years that is the average for Arkansas. This study investigates the sociodemographic, behavioral and health-related data of 331 adults experiencing homelessness in Central Arkansas. Data was collected by structured interviews during recent homeless counts. Participants ranged in age from 18 to 70, with quartiles established for ages 18-35, 36-45, 46-55 and 56 –70. Sociodemographic comparisons of the groups included education, income, months homeless, work and veteran status, behavioral variables included alcohol use and drug abuse, whereas health-related comparisons included reported diabetes, heart disease, diabetes, high blood pressure, heart disease, problems with hearing, vision, walking as well as mental health including anxiety and depression; in addition, medication procurement and available assistance from family and friends were compared. Preliminary results suggest significant differences, as well as some similarities, among the four age quartiles. Analyses are on-going.

